# A protocol of Chinese expert consensuses for the management of health risk in the general public

**DOI:** 10.3389/fpubh.2023.1225053

**Published:** 2023-09-28

**Authors:** Danni Wang, Qiangsheng He, Bin Xia, Jie Zheng, Wangnan Cao, Shaochen Su, Fulan Hu, Jiang Li, Yuelun Zhang, Zhengjia Ren, Xue Li, Xinyin Wu, Yafang Huang, Yongjiang Tang, Fuxin Wei, Huachun Zou, Huaili Jiang, Junjie Huang, Wenbo Meng, Ming Bai, Kehu Yang, Jinqiu Yuan, on behalf of the Chinese Health RIsk MAnagement Collaboration (CHRIMAC)

**Affiliations:** ^1^Department of Epidemiology and Biostatistics, Clinical Big Data Research Center, The Seventh Affiliated Hospital, Sun Yat-sen University, Shenzhen, China; ^2^Department of Endocrine and Metabolic Diseases, Shanghai Institute of Endocrine and Metabolic Diseases, Ruijin Hospital, Shanghai Jiao Tong University School of Medicine, Shanghai, China; ^3^Shanghai National Clinical Research Center for Metabolic Diseases, Key Laboratory for Endocrine and Metabolic Diseases of the National Health Commission of the PR China, Shanghai Key Laboratory for Endocrine Tumor, State Key Laboratory of Medical Genomics, Ruijin Hospital, Shanghai Jiao Tong University School of Medicine, Shanghai, China; ^4^MRC Integrative Epidemiology Unit (IEU), Bristol Medical School, University of Bristol, Oakfield House, Oakfield Grove, Bristol, United Kingdom; ^5^Department of Social Medicine and Health Education, School of Public Health, Peking University, Beijing, China; ^6^Healthy Examination & Management Center, The First Hospital of Lanzhou University, Lanzhou, China; ^7^Department of Epidemiology and Biostatistics, School of Public Health, Shenzhen University Health Science Center, Shenzhen, China; ^8^Office for Cancer Screening, National Cancer Center, National Clinical Research Center for Cancer, Cancer Hospital, Chinese Academy of Medical Sciences and Peking Union Medical College, Beijing, China; ^9^Chinese Academy of Medical Sciences Key Laboratory for National Cancer Big Data Analysis and Implement, Beijing, China; ^10^Medical Research Center, Peking Union Medical College Hospital, Chinese Academy of Medical Sciences and Peking Union Medical College, Beijing, China; ^11^Department of Clinical Psychology, The Third Affiliated Hospital of Chongqing Medical University, Chongqing, China; ^12^Center for Safe Medication Practice and Research, Department of Pharmacology and Pharmacy, Li Ka Shing Faculty of Medicine, The University of Hong Kong, Hong Kong, China; ^13^Laboratory of Data Discovery for Health (D24H), Hong Kong Science Park, Hong Kong, China; ^14^Department of Medicine, School of Clinical Medicine, Li Ka Shing Faculty of Medicine, University of Hong Kong, Hong Kong, China; ^15^Department of Epidemiology and Health Statistics, Xiangya School of Public Health, Central South University, Changsha, China; ^16^School of General Practice and Continuing Education, Capital Medical University, Beijing, China; ^17^Department of Respiratory and Critical Care Medicine, West China Hospital, Sichuan University, Chengdu, China; ^18^Department of Orthopaedic Surgery, The Seventh Affiliated Hospital of Sun Yat-sen University, Shenzhen, China; ^19^Department of Epidemiology, School of Public Health (Shenzhen), Sun Yat-sen University, Shenzhen, China; ^20^Cancer Center, Zhongshan Hospital, Fudan University, Shanghai, China; ^21^Jockey Club School of Public Health and Primary Care, Faculty of Medicine, The Chinese University of Hong Kong, Hong Kong, China; ^22^Department of General Surgery, The First Hospital of Lanzhou University, Lanzhou, China; ^23^The Department of Nephrology, Xijing Hospital, The Fourth Military Medical University, Xi’an, China; ^24^Health Technology Assessment Center, Evidence Based Social Science Research Center, School of Public Health, Lanzhou University, Lanzhou, China; ^25^Evidence Based Medicine Center, School of Basic Medical Sciences, Lanzhou University, Lanzhou, China; ^26^Key Laboratory of Evidence Based Medicine and Knowledge Translation of Gansu Province, Lanzhou, China

**Keywords:** non-communicable diseases (NCDs), primary prevention, risk factors, risk management, protocols & guidelines

## Abstract

**Introduction:**

Non-communicable diseases (NCDs) represent the leading cause of mortality and disability worldwide. Robust evidence has demonstrated that modifiable lifestyle factors such as unhealthy diet, smoking, alcohol consumption and physical inactivity are the primary causes of NCDs. Although a series of guidelines for the management of NCDs have been published in China, these guidelines mainly focus on clinical practice targeting clinicians rather than the general population, and the evidence for NCD prevention based on modifiable lifestyle factors has been disorganized. Therefore, comprehensive and evidence-based guidance for the risk management of major NCDs for the general Chinese population is urgently needed. To achieve this overarching aim, we plan to develop a series of expert consensuses covering 15 major NCDs on health risk management for the general Chinese population. The objectives of these consensuses are (1) to identify and recommend suitable risk assessment methods for the Chinese population; and (2) to make recommendations for the prevention of major NCDs by integrating the current best evidence and experts’ opinions.

**Methods and analysis:**

For each expert consensus, we will establish a consensus working group comprising 40–50 members. Consensus questions will be formulated by integrating literature reviews, expert opinions, and an online survey. Systematic reviews will be considered as the primary evidence sources. We will conduct new systematic reviews if there are no eligible systematic reviews, the methodological quality is low, or the existing systematic reviews have been published for more than 3 years. We will evaluate the quality of evidence and make recommendations according to the GRADE approach. The consensuses will be reported according to the Reporting Items for Practice Guidelines in Healthcare (RIGHT).

## 1. Introduction

Non-communicable diseases (NCDs), also known as chronic diseases, represent the leading cause of mortality and disability globally. In 2019, NCDs were responsible for 74% of all deaths and 63.8% of disability-adjusted life years (DALYs) worldwide (1). Prevention and control of NCDs have been emphasized in the United Nations’ 2030 Agenda for Sustainable Development (2). In 2015, all United Nations Member States committed to reducing a third of premature NCD mortality by 2030 (2). Nevertheless, achieving this goal is a great challenge for China. According to the Report on the Nutrition and Chronic Diseases Status of Chinese Residents, in 2019, more than 88.5% of the deaths in China were attributed to NCDs, of which 80.7% were due to cardiovascular diseases (CVDs), cancers, chronic respiratory diseases and diabetes (3). Although premature mortality from NCDs decreased from 18.5% in 2015 to 16.5% in 2019, there is still a long way towards achieving the 2030 agenda goals (3). A report published by the World Bank showed that the number of people suffering from at least one NCD in China would increase explosively, and the estimated number of NCD patients would increase by 101.7 million from 2010 to 2030 (4).

Confronting the increasing threats from NCDs, guidelines already released based on the NCD risk factors cannot fully satisfy the health demand of the general Chinese population. Robust evidence has demonstrated that modifiable behavioural risk factors such as unhealthy diet, smoking, alcohol consumption and physical inactivity are the leading causes of NCDs (5). In China, numerous guidelines have been published to improve health behaviour in the general population (6–8). For example, the Chinese Nutrition Society released and updated five editions of the Chinese Dietary Guidelines from 1989 to 2022 (9). However, improvement in lifestyle behaviours is slow, and some even turn worse in Chinese. For instance, the intakes of processed meat, red meat, and sugar-sweetened beverage showed increasing trends over time in the past decades (10). Several reasons hinder the general population from using available guidelines to achieve a healthy lifestyle. Firstly, overall health literacy is relatively low among the Chinese population, and most people are unaware of their own risks of suffering from NCDs, so they would not actively acquire healthcare-related knowledge (11). Secondly, most guidelines were clinical practice guidelines targeting clinicians rather than the general population. Last, current guidelines or consensuses generally provide “undifferentiated” recommendations, making it difficult for individuals to adopt prevention measures according to their own health conditions and lifestyle (12).

Personalized health risk management by evaluating disease susceptibility and tailoring preventive intervention strategies for individuals can promote health in a more cost-effective way (13). Health risk assessment (HRA) is the core step for implementing personalized health risk management. HRA can systematically collect personal health information, assess disease risks, and provide users with individualized feedback while linking the individuals with follow-up health promotion interventions (14). The complete HRA process (risk assessment, tailored feedback, and management) has been proven to be effective in improving health (15). According to a study by Shekelle et al., HRA programmes could improve health behaviour (especially exercises), physiological indicators (especially diastolic blood pressure and weight), and general health status (15). A prospective study from the Netherlands showed that voluntary participation in a Web-based HRA with tailored feedback at a worksite reduced CVD risk by nearly 18% among participants at high CVD risk and by nearly 5% among all participants after 7 months (16). Considering the benefits of HRA in promoting health, the Affordable Care Act (ACA, Section 4103) in the United States requires that HRA and subsequent tailored risk management strategies should be applied to Medicare beneficiaries (17). However, in China, due to incomplete data on risk factors related to NCDs, a standardized HRA index system and an assessment tool that can be generalized nationwide have not yet been established (18).

There are still many barriers to implementing HRA programmes in China. First, health management, particularly for health risks, is a relatively new area in China (12). There is an urgent need for health specialists, government investment, and research in this field. Second, for most of the general population, medical treatment is the mainstream and prevention is often not considered equally important (19). Third, comprehensive, evidence-based guidance for the risk management of major NCDs for the general Chinese population has not been established.

In order to promote the prevention of major NCDs in the general population, we plan to develop a series of expert consensuses on health risk management targeting the general population in China. The objectives of these consensuses are (1) to identify and recommend suitable risk assessment methods for the Chinese population; and (2) to make recommendations for the prevention of major NCDs by integrating the current best evidence and experts’ opinions.

## 2. Methods

This series of expert consensuses will be developed by the Chinese Health Risk Management Collaborative (CHRIMAC), which is an academic cooperative group of medical specialists with common interests in health risk assessment and prevention. CHRIMAC now includes over 80 members from epidemiology, healthcare management, clinical medicine, nutriology, and family medicine from 35 well-known medical institutions such as the affiliated hospitals of Sun Yat-sen University, Fudan University, The Chinese University of Hong Kong, Hong Kong University, Lanzhou University, etc. The inclusion of these medical institutions does not adopt a randomized approach, but is based on convenience – there is already a good foundation for cooperation among medical institutions. CHRIMAC will dynamically include experts from multiple disciplines in the future to develop the CHRIMAC expert consensus series. CHRIMAC is planning to develop consensuses covering major NCDs. In the first stage, we will focus on 15 diseases, including stroke, ischemic heart disease, diabetes mellitus, dementia, chronic kidney disease (CKD), chronic obstructive pulmonary disease (COPD), osteoporosis, lung cancer, breast cancer, gastric cancer, colorectal cancer, oesophageal cancer, liver cancer, pancreatic cancer, and cholelithiasis. The selection of these 15 diseases is mainly based on expert discussion. Three aspects were considered during the expert discussion (1) whether the disease is common; (2) whether the disease is preventable; and (3) whether the included medical institutions have an advantage in preventing the disease. Because the methods for developing these consensuses are similar, we will not publish separate protocols for individual NCDs.

**Figure 1:**
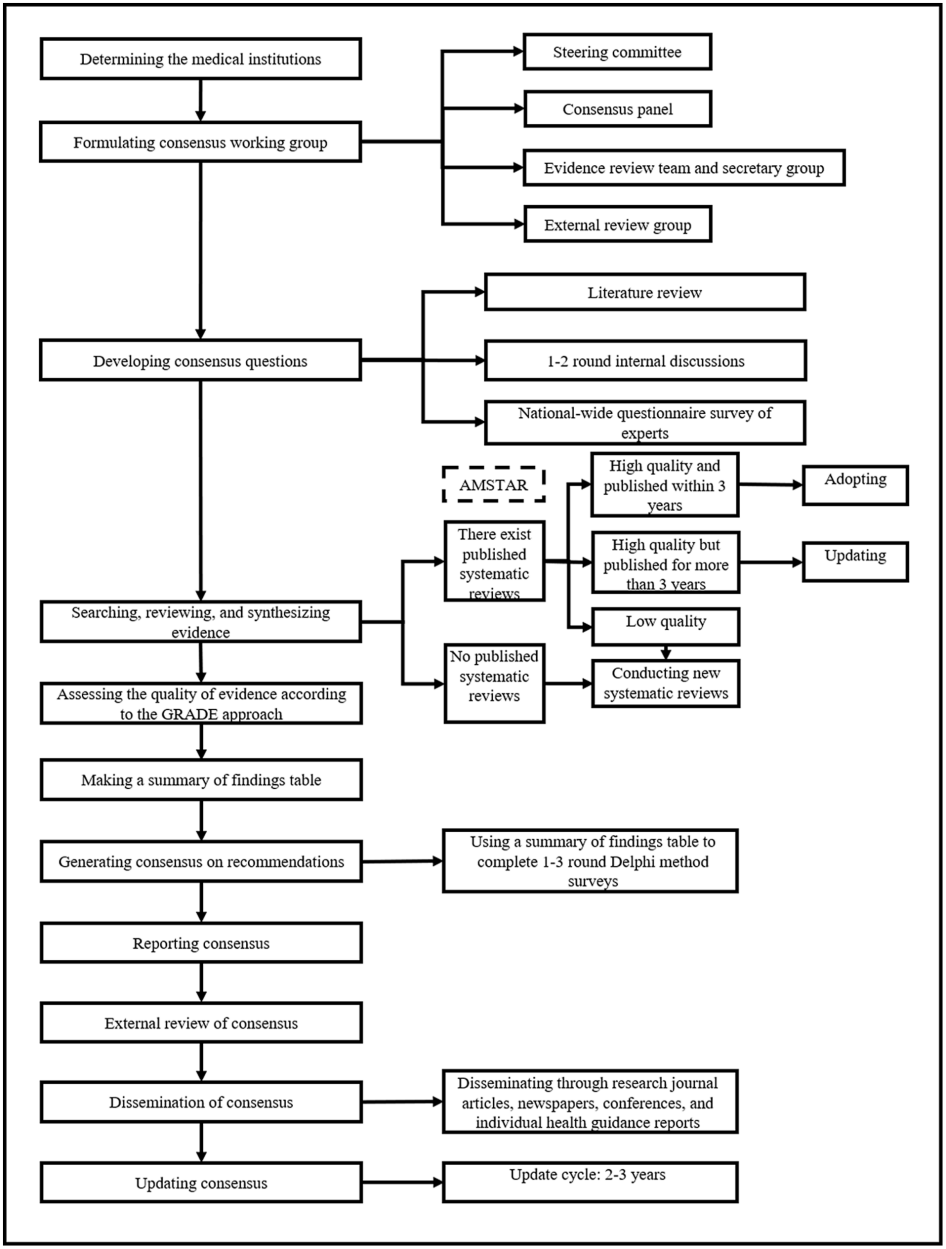
Flowchart of consensus development.

The overall methodology and process of the expert consensuses will be guided by the WHO handbook for guideline development (2014 edition) and the Chinese Guidance for Development/Updating Clinical Practice Guidelines (2022 edition) (21, 22). Each consensus will be registered on the International Practice Guidelines Registry Platform (IPGRP). A flowchart of the development process for individual consensus is shown in Figure 1.

The consensuses will be applied to hospitals at all levels, community healthcare centers, health management centers (this type of healthcare institution can formulate personalized physical examination packages according to the different demands of clients, assess clients’ health risks, establish exclusive health records, and propose personalized health management strategies with targeted recommendations), centers for disease control and prevention, and other healthcare institutions. The target audience of these consensuses is the general population in all regions of China, especially adults at high risk of major NCDs.

### 2.1. Consensus working group

The consensus working group will consist of the steering committee, consensus panel, evidence review team, external review group, and secretary group. All working group members will complete the conflict-of-interest disclosure form, update the conflict-of-interest situation promptly during the consensus development process, and release the disclosure form when the consensus is published.

#### 2.1.1. Steering committee

The steering committee will consist of 4–5 leading experts on the specific NCD and guideline methodologists. The primary responsibilities of the committee are to (1) determine the theme and scope of the consensuses; (2) establish other working groups and manage their conflicts of interest; (3) approve the consensus proposal; (4) supervise the consensus development process; (5) approve the recommendation statements and the full text of the consensuses; and (6) monitor and evaluate the implementation of the consensuses.

#### 2.1.2. Consensus panel

The consensus panel will be comprised of 20–30 experts, with one chairman and 1–2 vice-chairman. The experts should be representative in terms of region and specialty (clinical medicine, preventive medicine, healthcare management, guideline methodology). The panel will include 3–4 economists, patient representatives, and public representatives to balance cost-effectiveness and social justice. The primary responsibilities of the consensus panel are to (1) determine the final consensus questions; and (2) determine the strength of the recommendation statements.

#### 2.1.3. Evidence review team and secretary group

The evidence review team will include 10–15 master/PhD students or research assistants with systematic training in Evidence-Based Medicine. They will work collaboratively in coordination and management of consensus development, searching for evidence, evaluating and synthesizing evidence, and producing GRADE grids. The secretary group will be responsible for arranging and recording all work, liaising and communicating with consensus experts and addressing other matters which are not covered by other working groups. The leader of the evidence review team will also be the primary coordinator of this consensus.

#### 2.1.4. External review group

The external review group will include 5–8 experts in the related field who are not involved in the development of the consensuses. They will review and comment on the first draft of the consensuses. To increase the readability of the consensuses for the general population, we will invite 3–5 people with various occupation backgrounds and education levels to review the full text before publication.

### 2.2. Development of consensus questions

Consensus questions will be initially proposed by the steering committee based on their expertise, literature review, and 1–2 rounds of internal discussions. Then, an online questionnaire survey of 100–200 clinicians, healthcare managers and community health workers across China will be carried out to vote on the importance of consensus questions and collect additional consensus questions. Subsequently, the consensus panel will vote on the final questions through a consensus process of iterative discussions with all members of the consensus panel. For each candidate question, the importance will be graded from 1 (unimportant, should not be included) to 7 points (utmost importance, should be included). A question will be included if >75% of the participants vote 6 or 7 points for importance.

### 2.3. Evidence search

For consensus questions, the evidence review team will first search for relevant consensus guidelines and systematic reviews in PubMed, EMBASE, Cochrane Library, Wanfang, and CNKI. The search will not stop being updated until 6 months before the official publication of the consensuses. We will also search clinical guidelines websites, including Medlive, National Guideline Clearinghouse (NGC), Guideline International Network (GIN) and World Health Organization (WHO), for relevant guidelines/consensuses. Web search engines such as Google Scholar will be considered as additional sources of evidence.

### 2.4. Systematic reviews

We will consider systematic reviews and meta-analyses as the primary evidence sources for each consensus question. The evidence review team will conduct new systematic reviews/meta-analyses if (1) there is no eligible systematic review or meta-analysis; (2) the methodological quality of existing systematic reviews or meta-analyses is low; and (3) the existing systematic reviews or meta-analyses have been published for more than 3 years.

The evidence review team will carry out rapid systematic reviews according to the Cochrane Handbook for Systematic Reviews of Interventions (22). Briefly, new systematic reviews will include randomized controlled trials (RCTs) or observational studies (cohort study and case–control study) for the individual consensus questions. The evidence review team will develop search strategies based on the PICO framework (Participant, Intervention/Exposure, Comparison, and Outcome) and then carry out an electronic search for the aforementioned databases. Meanwhile, conference abstracts and references of included original papers will also be searched for additional eligible studies. For all potentially eligible studies, the review team will exclude duplicate records by EndNote and then eliminate irrelevant studies by reviewing the titles, abstracts and full texts. The methodological quality of included studies will be evaluated with the Cochrane risk-of-bias tool (for RCTs) and Newcastle-Ottawa Scale (NOS) (for observational studies) (23, 24). The study inclusion, data extraction, and quality assessment will be independently completed by two team members and crossly checked. Discrepancies will be solved by discussing or consulting a third-term member. The clinical, methodological, and statistical heterogeneity of included studies will be assessed. When appropriate, the effects will be pooled with a random-effect model. A Funnel plot will be applied to evaluate publication bias.

### 2.5. Assessment of the quality of evidence

For each consensus question, the quality of evidence will be evaluated based on the combined effects of systematic reviews or meta-analyses. According to the GRADE approach (25, 26), the quality of evidence will be classified as high, moderate, low, and very low (Table 1). Evidence from RCTs and observational studies will be regarded as high and low quality, respectively. Meanwhile, the quality of evidence will be downgraded for five factors – study limitations (risk of bias), imprecision, inconsistency, indirectness, and publication bias, and be upgraded for three factors – large or very large effect, dose–response relation, and plausible residual confounding. The best available body of evidence related to consensus questions will be presented with a summary of the finding table.

**Table 1:** Quality of evidence grades.

Grade	Definition
High	We are very confident that the true effect lies close to that of the estimate of the effect.
Moderate	We are moderately confident in the effect estimate: the true effect is likely to be close to the estimate of the effect, but there is a possibility that it is substantially different.
Low	Our confidence in the effect estimate is limited: the true effect may be substantially different from the estimate of the effect.
Very low	We have very little confidence in the effect estimate: the true effect is likely to be substantially different from the estimate of effect.

### 2.6. Consensus process

The steering committee, with assistance from the evidence review team, will discuss and make one or more preliminary recommendation statements for each consensus question. Then, the consensus panel will vote for the strength of the preliminary recommendation statements by Delphi methods. The preliminary recommendation statements, the evidence profile and other materials will be sent to the consensus group 1–2 weeks before the consensus meeting. The consensus experts will vote for the strength of the recommendations by considering four domains, namely estimates of effect for desirable and undesirable outcomes of interest, confidence in the estimates of effect, estimates of values and preferences, and resource use (26). The voting procedure will be implemented in two steps (26). First, the recommendation for or against a particular intervention should be approved by at least 50% of the panel, with less than 20% preferring the alternative. Failure to meet this criterion will lead to no recommendation. Second, a recommendation will be graded as strong rather than weak if at least 70% of the panel endorses it as strong. Strong recommendations will be made using the phrase “we recommend.” If this criterion is not met, the recommendation will be considered “weak” and expressed as “we suggest” (Table 2).

**Table 2:** The GRADE decision table for recommendations.

	Grade score of recommendations				
	1	2	0	−2	−1
Trade-offs of desirable and undesirable consequences of an intervention	Desirable clearly outweighs undesirable	Desirable probably outweighs undesirable	Trade-offs equally balanced or uncertain	Undesirable probably outweighs desirable	Undesirable clearly outweighs desirable
Strength of recommendation	Strong: “definitely do it”	Weak: “probably do it”	No explicit recommendation	Weak: “probably don’t do it”	Strong: “definitely don’t do it”
The result of the vote					

Firstly, determine the direction of recommendations. The recommendation for or against a particular intervention should be approved by at least 50% of participants, with less than 20% preferring the alternative. Failure to meet this criterion will lead to no recommendation.

Secondly, determine the strength of recommendations. A recommendation will be graded as strong rather than weak if at least 70% of participants endorse it as strong.

### 2.7. Consensus reporting

The consensus will be reported based on the Reporting Items for Practice Guidelines in Healthcare (RIGHT) (27). The initial manuscript will be drafted by the evidence review team and then sent to all consensus members for comments. Furthermore, the manuscript will be reviewed by the external review team. Finally, the recommendations will be reviewed and approved by the steering committee. The expert consensuses will be disseminated through research journal articles, newspapers, conferences, individual health guidance reports, and social media (including various forms such as images, audio and video).

### 2.8. Consensus updating and revising

The consensus will be updated and revised every 3 years in order to (1) address controversial content; (2) incorporate the latest evidence into the consensus; and (3) make adjustments to the recommendation statements based on the development of the specialty.

### 2.9. Patient and public involvement

This is a protocol for a series of expert consensuses, and patient and public involvement is not needed for the protocol.

## 3. Discussion

This is a protocol for the development of a series of expert consensuses, which will be used to establish comprehensive and evidence-based guidance for the risk management of 15 major NCDs in the Chinese population. Although China has already published several guidelines, these guidelines are mainly utilized in clinical settings and cannot satisfy the health demand of the general public. Additionally, the evidence for NCD prevention based on modifiable lifestyle factors in existing guidelines has still been disorganized. This series of expert consensuses can address the two problems.

Considering methodological aspects, this series of expert consensuses will incorporate specialists from multiple disciplines, including epidemiology, healthcare management, clinical medicine, nutriology, and family medicine, etc. Three significant strengths of the expert consensuses can be highlighted. First, under the guidance of the methodologists, each expert consensus will apply a comprehensive search strategy covering five electronic databases, four clinical guidelines websites and Google Scholar. Second, the latest systematic reviews and meta-analyses will be considered as the primary source of evidence. Third, recommendations will be made by integrating the current best evidence and experts’ opinions using the GRADE approach. Unfortunately, it is expected that most recommendations will be made based on observational studies, which will be the main limitation of our expert consensuses.

## Author contributions

JY and KY conceived and initiated this study. DW and JY drafted and finalized the content of the protocol. KY, XW, and YH provided methodological support for consensus development. QH, BX, JZ, WC, SS, FH, JL, YZ, ZR, XL, XW, YH, YT, FW, HZ, HJ, JH, WM, and MB critically revised the manuscript for important intellectual content and will provide their opinions in specific consensus based on their area of expertise. All authors contributed to the article and approved the submitted version.
